# Diseases of the Larynx

**Published:** 1912-05-18

**Authors:** 


### DISEASES OF THE LARYNX.

### The Cardinal Symptoms of Throat Disease.

The most important symptoms referable to disease of the throat fall under three headings: those affecting the functions (1) of respiration, (2) of phonation, and (3) of deglutition.

### Respiratory.

(1) Respiration may be obstructed in the larynx or in the trachea, and the symptoms of laryngeal and tracheal obstruction are usually readily distinguishable. In cases of laryngeal obstruction the patient usually sits with the head thrown well back in order to straighten the upper air-passage, and so to diminish as far as possible all resistance to the current of air. The stridor is loud, harsh, and inspiratory in character; indeed, the dyspnœa is altogether more marked during inspiration, and this is the case whether the obstruction is situated at the glottis or at the upper aperture of the glottis. This is due to the valve-like shape of the parts; the upper surface of the cords are flat, but below the narrowing of the glottis shelves away very gradually into the trachea, so that in the coronal section the cords somewhat resemble the cusps of the aortic valves. In a case of bilateral abductor paralysis, the cords may sometimes be seen to be sucked together during inspiration. In the same way the upper aperture tends to be drawn in by the inspiratory current, but offers less resistance to the expired air. In cases of laryngeal dyspnœa the entire larynx is sucked downwards during attempted inspiration, owing to the difference of pressure between the air above and below the obstruction, and this "laryngeal excursion" is an important sign of obstruction within the larynx. Finally, the voice is affected by nearly all causes of laryngeal obstruction; an important exception to this being bilateral abductor paralysis, where the cords remain in position for phonation, and the voice, though breathless, is not hoarse.

On the other hand, when the obstruction is situated within the trachea the patient assumes a position in which the head is bent forward in order to avoid stretching, and so further narrowing, the trachea. The stridor is less loud and is both expiratory and inspiratory, closely resembling the sound heard when a stethoscope is placed over the normal trachea. The "laryngeal excursion" is either absent or is much less marked, and the voice, though weak, is not otherwise affected.

### Phonatory.

(2) Though most laryngeal lesions affect the voice, the quality of the resulting hoarseness is often highly characteristic, and we can distinguish certain definite types which are distinctive of different forms of laryngeal disease. The harsh and raucous voice of laryngeal syphilis is the best known, and forms a complete antithesis to the weak aphonic voice of tuberculous laryngitis; to such an extent is this the case that it is of real help in the diagnosis where the laryngeal appearances are not decisive. The rough or gruff voice of a patient with a laryngeal neoplasm is also sufficiently characteristic to the practised ear.

### Aphonic Predisposition.

In cases of paralysis of one vocal cord a peculiar "diphonic" quality is sometimes apparent; the tension of the two cords being unequal, two different tones become audible in the voice at the same time. Any considerable experience will show that aphonia is not entirely dependent on the extent of the laryngeal lesion, but is largely proportionate to the lowering of the patient's neuro-muscular tone; extensive syphilitic ulceration does not destroy phonation in an otherwise healthy patient, whereas many elderly ladies habitually become aphonic with the least cold. General debility predisposes strongly to aphonia, and this explains the fact that so many sufferers from tuberculous laryngitis are aphonic, although the local lesions may be very slight; indeed, many chronic consumptives suffer from aphonia, although their larynges show no tuberculosis, but only a simple catarrh caused by cough and the irritation of the sputum. Functional aphonia, which is caused by insufficient action of the adductors and tensors of the cords, is a symptom of debility far more often than of hysteria, and not infrequently accompanies phthisis; the lungs should be carefully examined in every case of persistent functional aphona. In hysterical aphonia the voice usually comes and goes with great suddenness, and, when present, is quite strong and free from hoarseness.

Allusion may be made to disturbances of the voice caused by alterations of the vocal function of the nose. Normally, part of the air expired during phonation should pass through the nose, which imparts resonance to the vibrations; but it can be shut off at will by the elevation of the soft palate. Two abnormalities occur: either the air cannot pass through the nose, or else it cannot be shut off; some confusion arises because the laity term both these defects "speaking through the nose," but the two are really contrasted. The former is called "rhinolalia operta" and the latter "rhinolalia aperta" (closed and open nose speech). The closed is found in anyone with marked nasal obstruction; such a subject cannot speak the liquid consonants, for these require expiration through the nose, but says "b" for "m." Rhinolalia aperta is found in its most marked form in cases of cleft palate, and also in those who suffer from paresis of the soft palate, as after diphtheria; these people cannot pronounce the explosive consonants, for which the soft palate rises to cut off the nasal passages, and they say "m" instead of "b."

### Swallowing.

(3) The principal symptoms affecting deglutition are obstruction, pain, and regurgitation. Little need be said of the former two, except that they must be kept distinct in the mind. Swallowing may be prevented by a mechanical obstruction, which causes no pain (dysphagia), or it may be rendered impossible by pain without any real obstruction (odynphagia), as in cases of tuberculous laryngitis, when, if the pain can be overcome by local anodynes, delgutition can again be performed. Regurgitation may be nasal, laryngeal, or oral. Regurgitation into the nose occurs in cases of congenital defect, destruction, or paralysis of the palate. Regurgitation into the larynx sometimes occurs in cases of obstruction high up in the gullet, as at the back of the cricoid plate, when food collects above the stricture and overflows into the air-passage.

Food also tends to "go the wrong way" when infiltration of the upper aperture has fixed the arytenoids so that they cannot tilt forward and close the larynx during deglutition; it is not uncommon in late stages of tuberculous laryngitis. It is now well known that the epiglottis plays no part in preventing the entrance of food into the larynx. In the rare cases of laryngeal anæsthesia great danger arises from the entrance of food, and death usually ensues from deglutition-pneumonia. In the third form, food is regurgitated from the œsophagus into the mouth a variable time after it has been swallowed; it contains no hydrochloric acid and shows no signs of peptic digestion. The usual cause of this is dilatation of the œsophagus above a stricture, and this may reach an enormous size. A far rarer cause is the existence of a pharyngeal or œsophageal diverticulum. These conditions can usually be rendered apparent by skiagraphy after a bismuth meal and by the use of the direct œsophagoscope.

